# Food Swamps Surrounding Schools in Three Areas of Guatemala

**DOI:** 10.5888/pcd17.200029

**Published:** 2020-08-06

**Authors:** Aiken Chew, Alyssa Moran, Joaquin Barnoya

**Affiliations:** 1 Unidad de Cirugía Cardiovascular de Guatemala City, Guatemala, Guatemala; 2 Department of Health Policy and Management, Johns Hopkins University Bloomberg School of Public Health, Baltimore, Maryland; 3 Insituto de Investigación y Estudios Superiores en Salud, Universidad Rafael Landivar, Guatemala City, Guatemala

**Figure:**
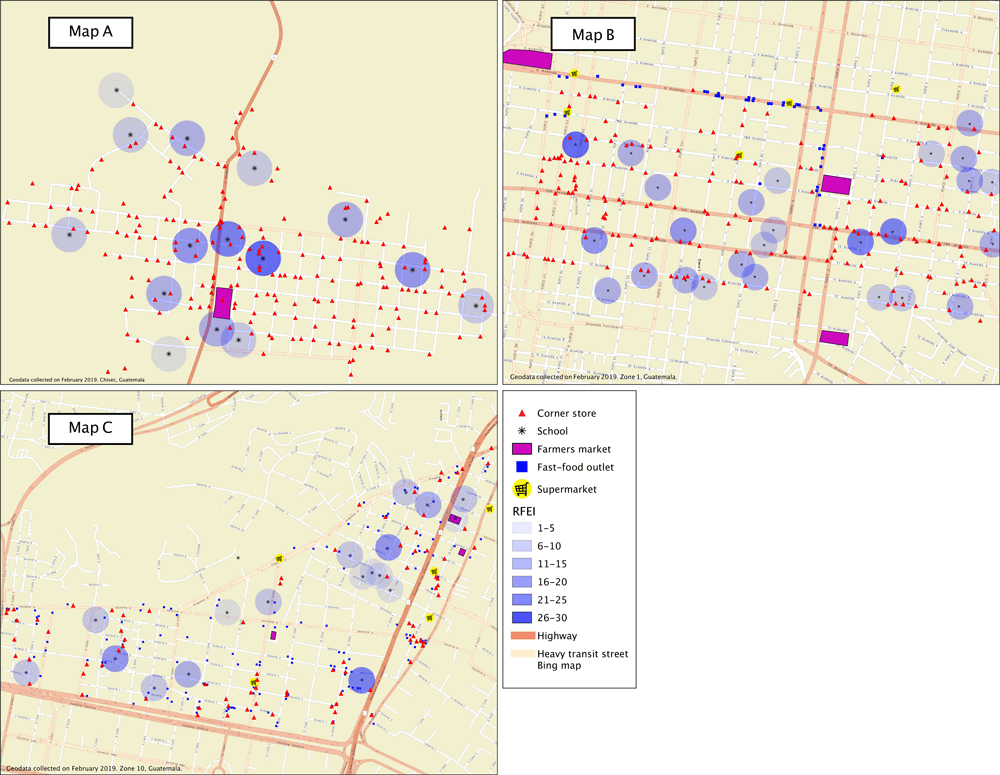
Retail food environment index (RFEI) (1) for 3 neighborhoods of different socioeconomic status in Guatemala: A, Chisec, a rural indigenous community located 4 hours north of Guatemala City; B, a middle-socioeconomic–status urban area of Guatemala City; and C, a high-socioeconomic–status urban area of Guatemala City. RFEI is the ratio of unhealthy to healthy food outlets: the higher the score, the less healthy the food environment. Maps identify stores — corner stores, fast-food outlets, farmers markets, and supermarkets — within a 150-meter radius of schools. All schools were located in food swamps (RFEI >3.89 = food swamp), defined as the mean RFEI across counties in the United States (2). Guatemala, September 2018.

## Background

Food swamps are environments saturated with unhealthy foods because of the large numbers of corner stores and fast-food outlets in them (2). In the United States, food swamps are defined as areas with 4 or more corner stores within 0.4 km (0.25 miles) of home or where the ratio of unhealthy to healthy food establishments exceeds 3.89 (2,3). In the United States, low-income and racial/ethnic minority populations are more likely than high-income white populations to live in food swamps (4). Living in a food swamp has been associated with unhealthy dietary behaviors and obesity among adults and young adolescents (3). Consequently, some US municipalities have adopted zoning or permitting laws to improve the food environment and reduce disparities in obesity prevalence (2,3).

In Latin America, overweight and obesity increased in the last decade. Guatemala, in particular, is struggling with the double burden of disease where underweight and overweight coexist (5). In 2015, 28% of Guatemalan students aged 13 to 17 years were overweight and 7% were obese. Most students (65%) reported drinking carbonated soft drinks at least once a day (6). Much of the rising prevalence in overweight and obesity has been attributed to the growing predominance of unhealthy packaged and fast foods (7). Corner stores in Guatemala sell primarily energy-dense, nutrient-poor snacks and sugary drinks, which are heavily marketed to and consumed by children (8–10). In addition, fast-food outlets in Guatemala City have been found to target children by using price incentives and toy giveaways (11). Public health action to improve the food environment has been limited. Our maps illustrate the prevalence of food swamps and disparities in exposure to unhealthy food environments by socioeconomic status (SES) and urbanicity as a tool to influence Guatemalan public health policy.

## Data sources and map logistics

We mapped the food environment in 3 neighborhoods, 1 high-SES and 1 middle-SES neighborhood in urban Guatemala City and 1 in Chisec, a low-SES rural indigenous community 4 hours north of Guatemala City. Thresholds for SES were based on the average unit price of land in 2017 (12). A trained research assistant walked through each neighborhood in September 2018. Corner stores, chain supermarkets, farmers markets, fast-food outlets, and schools (private and public) were georeferenced with a GPS device (Garmin Oregon). We used QGIS version 2.18 (https://qgis.org/en/site/about/index.html), a free and open source geographic information program, to overlay locations of food outlets on a Bing (Esri) basemap layer. By using the “fixed distance buffer” analysis function from QGIS, we drew a 150-meter (0.09 miles) buffer around each school. This buffer was selected on the basis of conversations with school principals, who considered this a reasonable distance for children aged 4 to 12 years to walk to and from school (8). The “count points on polygons” function was used to assess the number of food outlets within the buffer. We used the retail food environment index (RFEI) to calculate the healthfulness of the food environment around each school (13). RFEI considers fast-food retailers and corner stores as unhealthy food outlets and supermarkets and farmers markets as healthy. The index is calculated as the ratio of unhealthy to healthy outlets: the higher the score, the less healthy the environment. Where we found no healthy outlets, we used 1 as the denominator. The RFEI of each school across the neighborhood was added and then divided by the number of schools in each territory to determine the mean RFEI.

## Highlights

We identified 280 corner stores (Table) in the 2 neighborhoods in Guatemala City and 204 in Chisec. Corner store density was higher in the middle-SES urban neighborhood and in rural Chisec; fast-food restaurant density was higher in the high-SES urban neighborhood. By using the RFEI cutoff of 3.89 (2), all surveyed neighborhoods were classified as food swamps. We found the highest RFEI in the middle-SES urban neighborhood and in rural Chisec.

**Table:** Number of Corner Stores, Fast-Food Outlets, and Schools in 3 Neighborhoods^a^, Guatemala, 2018

Characteristic	Neighborhood Socioeconomic Status		
	Urban		Rural
	Middle	High	Low
**Neighborhood area, mi^2^**	1.03	1.27	0.98
**Schools and food outlets, number (number per mi^2^)**			
Schools	26 (25)	19 (15)	15 (15)
Corner stores^b^	193 (187)	87 (68)	204 (208)
Fast-food outlets^c^	42 (41)	60 (47)	0
Supermarkets^d^	5 (5)	5 (4)	0
Farmers markets^e^	4 (4)	3 (2)	1 (1)
**Median (range) number of outlets within each school buffer^f^**			
Corner stores	11 (10–12)	3 (1-5)	11 (9.5–17)
Fast food	0	5 (2.5–9)	0
Farmers markets	0	0	0
Supermarkets	0	0	0
Schools with no store within the buffer	0	1	0
Mean Retail Food Environment Index^g^	12.6	8.3	12.9

a One high- and 1 middle-socioeconomic status neighborhood in urban Guatemala City and 1 low-socioeconomic status neighborhood in rural Chisec, Guatemala.

b Stores with sufficient product types to complete a fill-in or quick, single-meal shopping trip, often located as a storefront within the primary residence, usually part of the informal economy.

c Stores that prepare food in a few minutes and have no table service; includes to-go meals (eg, McDonald´s, Pizza Hut, Pollo Campero).

d Self-service shopping stores (usually chains) with a large variety of products, including fresh produce.

e Municipal outlets that mostly provide fresh produce from local farmers.

f Buffer of 150 meters (0.09 miles) around schools.

g The ratio of unhealthy to healthy food outlets: the higher the score, the less healthy the environment.

To our knowledge, ours is the first evidence of a food swamp in a low- and middle-income country, Guatemala. Our findings should be interpreted in light of some limitations. Because of time and financial constraints, the number of surveyed areas was limited. However, we were able to obtain comprehensive GIS data for corner stores, which often are not registered in the formal economy and likely contribute substantially to the intake of unhealthy foods and beverages.

## Action

Our maps show the proliferation of food swamps in Guatemala and suggest that approaches for creating healthier food environments vary by neighborhood. Although all neighborhoods had high RFEI scores, we found higher RFEI scores in Chisec and in the middle-income neighborhood of Guatemala City than in the high-income neighborhood of Guatemala City. In Chisec and in the middle-income neighborhood of Guatemala City, the higher RFEI was driven by the relatively large number of corner stores and lack of supermarkets. In the higher-income neighborhood of Guatemala City, RFEI was driven by the relatively large number of fast-food outlets. In Guatemala, these food outlets pose different regulatory challenges. For example, supermarkets and fast-food outlets are part of the formal economy, requiring a license to operate and primarily serving high- and middle-income consumers. Although these establishments could, theoretically, be regulated through zoning and licensing policies, lobbying and marketing from the food and beverage industry often halt any proposed government action. By contrast, most corner stores operate within the informal economy – businesses unregulated by the government. Informal businesses are more common in low-income areas of the country where formal employment is unavailable and thus serve mainly mid- to low-income consumers (10). These outlets are difficult to monitor and regulate but could be improved through local partnerships with store owners that increase the availability, affordability, and marketing of healthy foods in these outlets, similar to healthy corner store initiatives in the United States.

To date, no one has examined the healthfulness of the built environment in Guatemala across neighborhoods or explored sustainable, large-scale strategies to limit exposure to food swamps. A large number of businesses that sell unhealthy food and stark disparities exist across neighborhoods and should be monitored over time. Although we assessed only 3 neighborhoods in Guatemala, similar maps could be useful for engaging planners, developers, and policy makers in discussions around the built environment and health. Maps may also be useful to school administrators, who might adopt institutional policies (eg, nutritional standards) for food vendors to encourage students to eat on campus, thereby reducing students’ exposure to unhealthy retailers.
